# Smoking and subsequent risk of leukemia in Japan: The Japan Public Health Center-based Prospective Study

**DOI:** 10.1016/j.je.2016.07.005

**Published:** 2017-04-08

**Authors:** Tomotaka Ugai, Keitaro Matsuo, Norie Sawada, Motoki Iwasaki, Taiki Yamaji, Taichi Shimazu, Shizuka Sasazuki, Manami Inoue, Shoichiro Tsugane, the Japan Public Health Center-based Prospective Study Group

**Affiliations:** a Division of Molecular and Clinical Epidemiology, Aichi Cancer Center Research Institute, Nagoya, Japan; b Division of Hematology, Saitama Medical Center, Jichi Medical University, Omiya, Japan; c Department of Epidemiology, Nagoya University Graduate School of Medicine, Nagoya, Japan; d Epidemiology and Prevention Group, Center for Public Health Sciences, National Cancer Center, Tokyo, Japan; e Graduate School of Medicine, The University of Tokyo, Tokyo, Japan

**Keywords:** JPHC study, Prospective cohort study, Smoking, Leukemia

## Abstract

**Background:**

Cigarette smoking has been reported to be associated with an increased risk of leukemia. Most epidemiological evidence on the association between cigarette smoking and leukemia risk is from studies conducted in Western populations, however, and evidence from Asian populations is scarce.

**Methods:**

We conducted a large-scale population-based cohort study of 96,992 Japanese subjects (46,493 men and 50,499 women; age 40–69 years at baseline) with an average 18.3 years of follow-up, during which we identified 90 cases of acute myeloid leukemia (AML), 19 of acute lymphoblastic leukemia (ALL), and 28 of chronic myeloid leukemia (CML). Hazard ratios (HRs) and 95% confidence intervals (CIs) were estimated using a Cox regression model adjusted for potential confounders.

**Results:**

When we adjusted for age, sex, and study area, our findings showed no significant association or increasing dose–response relationship between risk of AML and cigarette smoking overall. However, after further adjustment for body mass index and occupation, current smokers with more than 30 pack-years of cigarette smoking had a significantly increased risk of AML compared to never smokers among men (HR 2.21; 95% CI, 1.01–4.83). This increased risk was not clear among women.

**Conclusions:**

Our results suggest that cigarette smoking increases the risk of AML in Japanese men. The associations of smoking with AML among women, and with CML and ALL among men and women, should be assessed in future studies.

## Highlights

• The first report based on cohort study to evaluate smoking and leukemia risk in Japan.
• This study indicates that cigarette smoking increases the risk of AML among men.
• Larger studies are needed on the association of smoking with AML for women.
• Associations with CML and ALL for both sexes also need further study.

## Introduction

The association between cigarette smoking and risk of leukemia has been investigated in epidemiological studies. Recent meta-analyses showed that cigarette smoking is a significant risk factor for the development of acute myeloid leukemia (AML).^1^, ^2^ In 2004, the Surgeon General of the United States and the International Agency for Research on Cancer (IARC) reported that smoking is associated with an increased risk of AML,^3^, ^4^ while there is insufficient supporting evidence for the risk of lymphoid leukemia. Most epidemiological evidence on the association between cigarette smoking and leukemia risk has been obtained from studies in Western populations, however, and evidence from Asian populations is scarce.

Here, we investigated the effect of cigarette smoking on the risk of leukemia, including AML, acute lymphoblastic leukemia (ALL), and chronic myeloid leukemia (CML), in a large-scale population-based cohort study of a Japanese population.

## Methods

### Study population

The design of the Japan Public Health Center-based Prospective Study has been detailed elsewhere.^5^ Briefly, the study was launched in 1990 for cohort I and in 1993 for cohort II. Cohort I covered five prefectural public health center (PHC) areas (Iwate, Akita, Nagano, Okinawa, and Tokyo) and cohort II covered six areas (Ibaraki, Niigata, Kochi, Nagasaki, Okinawa, and Osaka). In the present analysis, we excluded all subjects in the Tokyo area because their incidence data were not available, and we also excluded some in the Osaka area because different definitions of the study population had been applied to them. A baseline survey was conducted in 1990 for cohort I and in 1993–1994 for cohort II, with a high response rate (81%). Participants with a self-reported history of cancer at baseline (*n* = 2302) were excluded. Subjects were followed from the date of the baseline survey to December 31, 2012. Residence status and survival were confirmed annually through the resident registry in each area, or, for those who had moved out of the study area, through the municipal office of the area to which they had moved. Data for subjects who had missing values for smoking status (*n* = 524) were excluded from this analysis, leaving a total of 96,992 subjects (46,493 men and 50,499 women) for analysis. The study protocol was approved by the institutional review boards of the National Cancer Center, Japan and Aichi Cancer Center Research Institute.

### Outcome

Study outcomes were defined as the incidence of newly occurring leukemia diagnosed during the study period. Leukemia was identified using active patient notification from major local hospitals in the study area and data collection from population-based cancer registries, with approval. We used death certificate files, with permission, as a supplement. The proportion of cases where incidence was ascertained using death certificate only was 15.8% for total leukemia and 6.7% for all types of cancer. Leukemia was coded using the International Classification of Diseases for Oncology, Third Edition (ICD-O-3). We defined the ICD-O-3 topology code C42.1, with morphology codes 9840, 9861, 9866, 9867, 9873, 9874, 9875, 9891 and 9896 as AML; 9875 and 9863 as CML; and 9832, 9833, 9834 and 9835 as ALL. Chronic lymphocytic leukemia (CLL), hairy cell leukemia, prolymphocytic leukemia, and adult T cell lymphoma/leukemia (ATL) were excluded because they were classified into mature lymphoid neoplasms in ICD-O-3. Chronic myelomonocytic leukemia was also excluded because it was classified into myelodysplastic/myeloproliferative diseases. The earliest date of diagnosis was used for patients who developed multiple primary neoplasms at different times. We coded cases as multiple primaries when both a chronic and an acute neoplasm were diagnosed simultaneously or within 21 days according to the rules of the Surveillance Epidemiology and End Results (SEER) Program.^6^

### Exposure data

Exposure data were based on a baseline self-administered questionnaire survey on various life-style factors, including smoking habit. Regarding smoking, subjects were categorized as never, former, or current smokers. In JPHC Study I, participants were asked whether they had ever smoked. If not, they were classified as never smokers. Ever smokers were further asked if they smoked at the time of the baseline survey. Those with positive and negative responses to the question were categorized into current smokers and former smokers, respectively. In JPHC Study II, participants were first asked whether they smoked at baseline. Subjects who reported smoking at baseline were defined as current smokers. Those who had quit smoking and did not smoke at baseline were requested to indicate the age at cessation, the number of cigarettes smoked per day during the smoking period, and the age at starting smoking. Those with or without responses to these questions were classified as former smokers or never smokers, respectively. Smoking intensity for current smokers and ever smokers was evaluated using pack-years, defined by multiplying the number of years of smoking by the number of cigarettes per day divided by 20. We classified current smokers using the two smoking intensity categories of <30 pack-years and ≥30 pack-years. We also classified ever smokers using the four smoking intensity categories of <10 pack-years, 10–19 pack-years, 20–29 pack-years, and ≥30 pack-years. Body mass index (BMI) was calculated from the self-reported height and weight by dividing the weight in kilograms by the square of height in meters.

### Statistical analysis

Person-years for leukemia incidence were accrued from the date of the baseline survey until the date of occurrence of leukemia, emigration from the study area, death, or end of the study period, whichever came first. Subjects lost to follow-up were censored at the last confirmed date of presence in the study area. Hazard ratios (HRs) and their 95% confidence intervals (CIs) were calculated using the Cox proportional hazards model as a measure of association between the risk of leukemia associated with smoking categories at baseline (never smokers, former smokers, pack-years in current smokers [<30 and ≥ 30 pack-years], and pack-years in ever smokers [<10, 10–19, 20–29 and ≥ 30 pack-years]). We set never smokers as the reference category. However, when we performed analysis of ALL, we set never and former smokers as the reference category because no male never smoker was diagnosed with ALL during the follow-up period. We estimated two types of HRs: 1) adjusted for age at baseline (continuous), sex, and study area (10 PHC areas); and 2) adjusted for age at baseline, sex, BMI (<18.5, 18.5–24.9, 25–29.9, and ≥30 kg/m^2^), occupation (professional or office worker, sales clerk or other, farmer, manual laborer, unemployed and missing), and study area (10 PHC areas). We considered occupation as an indicator of individual socioeconomic conditions. The P-values for trends were assessed by assigning ordinal variables in each category. All statistical analyses were done using Stata version 13.1 software (Stata Corp., College Station, TX, USA), with a P value < 0.05 considered to be statistically significant.

## Results

During 1,782,762 person-years of follow-up (average, 18.3 years) for the 96,992 subjects (46,493 men and 50,499 women), 90 AML cases (55 men and 35 women), 19 ALL cases (11 men and 8 women), and 28 CML cases (19 men and 9 women) were newly diagnosed. Baseline characteristics of study subjects according to smoking status are shown in Table 1. At baseline, 24.2%, 23.8%, and 52.1% of the men were never smokers, former smokers, and current smokers, respectively. In contrast, most of the women (92.4%) were never smokers, with only 1.5% and 6.1% being former smokers and current smokers, respectively. In pack-year categories, a smaller number of women were heavy smokers than men. Mean BMI was approximately 23 kg/m^2^ in each smoking category in both men and women. Distributions of occupation at baseline are also shown in Table 1.

**Table 1:** Baseline characteristics of study subjects by smoking status.

	Smoking status							
	Never	Former	Pack-years in current smoker		Pack-years in ever smoker			
			<30	≥30	<10	10–19	20–29	≥30
Men (n = 46,493)								
Person-years	205,317	194,378	190,611	230,695	60,254	96,230	150,189	309,351
Number of subjects	11,235	11,041	10,632	13,564	3270	5376	8394	18,218
Proportion, %	24.2	23.8	22.9	29.2	7.0	11.6	18.1	39.2
Mean (SD) age, years	52.0 (7.5)	53.9 (8.3)	48.6 (7.4)	53.1 (7.7)	49.9 (7.4)	50.1 (8.0)	49.7 (7.9)	54.0 (7.9)
Mean (SD) body mass index, kg/m^2^	23.5 (2.9)	23.2 (2.8)	22.6 (2.8)	22.7 (2.9)	23.1 (2.8)	22.8 (2.8)	22.7 (2.8)	22.9 (2.9)
Occupation, %								
Professionals and office workers	21.0	19.9	18.6	17.3	23.1	19.8	18.9	17.1
Sales clerk or others	20.7	22.5	23.4	23.7	23.4	22.9	24.0	23.0
Farmers	27.1	23.4	19.1	24.9	18.6	19.2	19.9	25.7
Manual laborers	24.4	23.9	32.8	27.0	28.6	31.0	30.8	25.3
Unemployed	5.6	9.2	4.8	5.7	5.2	5.9	5.4	7.5
Missing	1.2	1.1	1.3	1.4	1.2	1.2	1.0	1.4
Women (n = 50,499)								
Person-years	886,127	13,276	43,659	9126	27,937	17,490	9697	11,468
Number of subjects	46,662	742	2517	547	1543	1020	593	681
Proportion, %	92.4	1.5	5.0	1.1	3.1	2.0	1.2	1.4
Mean (SD) age, years	52.5 (8.0)	52.1 (8.6)	50.0 (7.7)	53.9 (8.1)	49.4 (7.6)	50.9 (8.2)	51.1 (7.9)	54.4 (8.2)
Mean (SD) body mass index, kg/m^2^	23.0 (3.1)	23.3 (3.4)	22.6 (3.5)	23.1 (3.8)	22.8 (3.4)	22.7 (3.7)	22.7 (3.4)	23.1 (3.7)
Occupation, %								
Professionals and office workers	11.3	9.4	11.1	6.6	12.0	10.8	8.9	5.9
Sales clerk or others	17.5	25.9	27.1	32.7	26.3	26.0	30.5	30.8
Farmers	23.8	11.3	8.4	10.2	10.0	7.9	7.3	11.0
Manual laborers	13.5	12.3	14.9	9.5	15.6	13.3	14.0	9.7
Unemployed	32.8	39.9	36.6	38.4	35.1	39.9	37.3	39.8
Missing	1.1	1.2	1.8	2.6	1.1	2.1	2.0	2.8

SD, standard deviation.

Table 2 shows the adjusted HRs for AML and CML by smoking category. When we adjusted for age, sex, and study area, we failed to observe a significant association or increasing dose–response relationship between AML risk and cigarette smoking overall. However, after further adjustment for BMI and occupation, current smokers with more than 30 pack-years of cigarette smoking had a significantly increased risk of AML compared to never smokers among men (HR 2.21; 95% CI, 1.01–4.83). This risk increase was not clear among women. An association with smoking was not apparent for the risk of CML among men, albeit the number of the subjects was small. No female smoker was diagnosed with CML during the follow-up period.

**Table 2:** Hazard ratios of acute myeloid leukemia and chronic myeloid leukemia according to smoking status.

	Smoking status										
	Never	Former	Pack-years in current smokers		P trend^e^	Never	Pack-years in ever smokers				P trend^f^
			<30	≥30			<10	10–19	20–29	≥30	
Acute myeloid leukemia											
Total											
Person-years	1,091,444	207,654	234,270	239,822		1,091,444	88,191	113,720	159,885	320,819	
Number of cases	42	17	8	23		42	5	7	7	29	
HR^a^ (95% CI)	1.00 (Reference)	1.64 (0.79–3.42)	0.85 (0.36–2.00)	1.89 (0.94–3.81)	0.197	1.00 (Reference)	1.43 (0.53–3.84)	1.40 (0.57–3.44)	0.97 (0.39–2.42)	1.64 (0.84–3.18)	0.208
HR^b^ (95% CI)	1.00 (Reference)	1.68 (0.80–3.50)	0.91 (0.39–2.16)	2.02 (1.00–4.06)	0.132	1.00 (Reference)	1.49 (0.56–4.00)	1.50 (0.61–3.69)	1.04 (0.41–2.60)	1.72 (0.89–3.35)	0.161
Men											
Person-years	205,317	194,378	190,611	230,695		205,317	60,254	96,230	150,189	309,351	
Number of cases	9	17	6	23		9	5	5	7	29	
HR^c^ (95% CI)	1.00 (Reference)	1.73 (0.77–3.92)	0.81 (0.28–2.29)	2.04 (0.94–4.46)	0.160	1.00 (Reference)	2.12 (0.71–6.36)	1.26 (0.42–3.78)	1.13 (0.42–3.07)	1.82 (0.85–3.89)	0.198
HR^d^ (95% CI)	1.00 (Reference)	1.78 (0.79–4.03)	0.90 (0.32–2.58)	2.21 (1.01–4.83)	0.095	1.00 (Reference)	2.19 (0.73–6.59)	1.37 (0.46–4.11)	1.24 (0.46–3.37)	1.92 (0.90–4.11)	0.152
Women											
Person-years	886,127	13,276	43,659	9126		886,127	27,937	17,490	9697	11,468	
Number of cases	33	0	2	0		33	0	2	0	0	
HR^c^ (95% CI)	1.00 (Reference)	NA	1.51 (0.36–6.38)	NA	0.986	1.00 (Reference)	NA	3.85 (0.91–16.2)	NA	NA	0.932
HR^d^ (95% CI)	1.00 (Reference)	NA	1.69 (0.40–7.20)	NA	0.881	1.00 (Reference)	NA	4.04 (0.95–17.3)	NA	NA	0.97
Chronic myeloid leukemia											
Total											
Person-years	1,091,444	207,654	234,270	239,822		1,091,444	88,191	113,720	159,885	320,819	
Number of cases	13	8	1	6		13	1	3	1	10	
HR^a^ (95% CI)	1.00 (Reference)	1.74 (0.57–5.32)	0.22 (0.03–1.86)	1.14 (0.35–3.78)	0.616	1.00 (Reference)	0.62 (0.08–5.09)	1.29 (0.32–5.22)	0.29 (0.03–2.49)	1.35 (0.46–3.94)	0.685
HR^b^ (95% CI)	1.00 (Reference)	1.72 (0.57–5.26)	0.23 (0.03–1.93)	1.19 (0.36–3.91)	0.676	1.00 (Reference)	0.63 (0.08–5.12)	1.34 (0.33–5.42)	0.30 (0.04–2.56)	1.37 (0.47–4.00)	0.662
Men											
Person-years	205,317	194,378	190,611	230,695		205,317	60,254	96,230	150,189	309,351	
Number of cases	4	8	1	6		4	1	3	1	10	
HR^c^ (95% CI)	1.00 (Reference)	2.01 (0.59–6.78)	0.29 (0.03–2.60)	1.34 (0.37–4.82)	0.833	1.00 (Reference)	0.89 (0.10–8.05)	1.67 (0.37–7.53)	0.36 (0.04–3.26)	1.60 (0.49–5.22)	0.532
HR^d^ (95% CI)	1.00 (Reference)	2.00 (0.59–6.75)	0.28 (0.03–2.59)	1.35 (0.37–4.87)	0.849	1.00 (Reference)	0.87 (0.10–7.83)	1.68 (0.37–7.58)	0.36 (0.04–3.24)	1.61 (0.49–4.26)	0.521

CI, confidence interval; HR, hazard ratio; NA, not available; PHC, public health center.

a HRs are adjusted for age at baseline (continuous), sex and study area (10 PHC areas).

b HRs are adjusted for age at baseline (continuous), sex, body mass index (<18.5, 18.5–24.9, 25–29.9, and ≥30 kg/m^2^), occupation (professional or office worker, sales clerk or other, farmer, manual laborer, unemployed, and missing), and study area (10 PHC areas).

c HRs are adjusted for age at baseline (continuous) and study area (10 PHC areas).

d HRs are adjusted for age at baseline (continuous), body mass index (<18.5, 18.5–24.9, 25–29.9, and ≥30 kg/m^2^), occupation (professional or office worker, sales clerk or other, farmer, manual laborer, unemployed, and missing), and study area (10 PHC areas).

e P-values for trends were calculated by assigning scores for categories of smoking status, with 1 for never smokers, 2 for former smoker, 3 for <30 pack-years, and 4 for ≥30 pack-years in current smokers.

f P-values for trends were calculated by assigning scores for categories of smoking status, with 1 for never smokers, 2 for <10 pack-years, 3 for 10–19 pack-years, 4 for 20–29 pack-years, and 5 for ≥30 pack-years in ever smokers.

Table 3 shows the adjusted HRs for ALL by smoking category. We also found no statistically significant association between smoking status and ALL risk among men. No female smoker was diagnosed with ALL during the follow-up period.

**Table 3:** Hazard ratios of acute lymphoblastic leukemia according to smoking status.

	Smoking status									
	Never and Former	Pack-years in current smokers		P trend^e^	Never and Former	Pack-years in current smokers				P trend^f^
		<30	≥30			<10	10–19	20–29	≥30	
Acute lymphoblastic leukemia										
Total										
Person-years	1,299,099	234,270	239,822		1,299,099	43,946	69,236	121,089	239,822	
Number of cases	11	5	3		11	1	2	2	3	
HR^a^ (95% CI)	1.00 (Reference)	2.07 (0.61–6.99)	1.25 (0.29–5.37)	0.614	1.00 (Reference)	2.36 (0.29–19.0)	2.80 (0.56–14.0)	1.51 (0.29–7.94)	1.23 (0.29–5.23)	0.654
HR^b^ (95% CI)	1.00 (Reference)	2.17 (0.64–7.36)	1.28 (0.30–5.49)	0.591	1.00 (Reference)	2.51 (0.31–20.3)	2.93 (0.58–14.7)	1.57 (0.30–8.29)	1.25 (0.29–5.35)	0.629
Men										
Person-years	399,695	190,611	230,695		399,695	24,230	54,057	112,325	230,695	
Number of cases	3	5	3		3	1	2	2	3	
HR^c^ (95% CI)	1.00 (Reference)	3.43 (0.78–15.1)	1.61 (0.32–8.02)	0.494	1.00 (Reference)	4.93 (0.49–49.2)	4.91 (0.80–30.3)	2.33 (0.37–14.6)	1.61 (0.32–8.00)	0.533
HR^d^ (95% CI)	1.00 (Reference)	3.43 (0.76–15.5)	1.60 (0.32–7.99)	0.513	1.00 (Reference)	5.02 (0.49–50.9)	4.99 (0.79–31.7)	2.33 (0.37–14.8)	1.59 (0.32–7.97)	0.556

CI, confidence interval; HR, hazard ratio; PHC, public health center.

a HRs are adjusted for age at baseline (continuous), sex, and study area (10 PHC areas).

b HRs are adjusted for age at baseline (continuous), sex, body mass index (<18.5, 18.5–24.9, 25–29.9, and ≥30 kg/m^2^), occupation (professional or office worker, sales clerk or other, farmer, manual laborer, unemployed, and missing), and study area (10 PHC areas).

c HRs are adjusted for age at baseline (continuous) and study area (10 PHC areas).

d HRs are adjusted for age at baseline (continuous), body mass index (<18.5, 18.5–24.9, 25–29.9, and ≥30 kg/m^2^), occupation (professional or office worker, sales clerk or other, farmer, manual laborer, unemployed and missing), and study area (10 PHC areas).

e P-values for trends were calculated by assigning scores for categories of smoking status, with 1 for never and former smokers, 2 for <30 pack-years, and 3 for ≥30 pack-years in current smokers.

f P-values for trends were calculated by assigning scores for categories of smoking status, with 1 for never and former smokers, 2 for <10 pack-years, 3 for 10–19 pack-years, 4 for 20–29 pack-years, and 5 for ≥30 pack-years in current smokers.

## Discussion

We investigated the association between smoking and the risk of leukemia in a population-based prospective study in Japan. The results showed that current smokers with more than 30 pack-years of cigarette smoking had a significantly increased risk of AML than never smokers in Japanese men, while associations with AML among women and ALL and CML among men and women were not clear.

Our suggested finding that smoking increases the risk of AML in Japanese men is consistent with the results of European studies.^1^, ^2^ A few epidemiological studies have evaluated the association between cigarette smoking and the risk of leukemia in Asia.^7^, ^8^, ^9^ A case–control study of 124 cases and 284 controls in Japan reported that cigarette smoking increased the risk of acute non-lymphocytic leukemia (ANLL), with an odds ratio (OR) for current smokers relative to never smokers of 1.76 (95% CI, 0.96–3.23).^8^ Another case–control study of 722 cases and 1444 controls in China also reported that cigarette smoking increased the risk of AML, with an OR relative to never smokers of 1.28 (95% CI, 1.00–1.63) for ever smokers.^9^ In contrast, a case–control study of 415 cases and 1700 controls in Korea reported no significant association between the risk of AML and ever-smoking (OR relative to never smokers, 0.98; 95% CI, 0.79–1.22).^10^ The only prospective cohort study, which involved 1,212,906 participants and 355 leukemia cases in Korea, reported no significant increased risk of leukemia overall among men, with HRs for current smokers relative to never smokers of 1.1 (95% CI, 0.8–1.5).^7^ Unfortunately, this study did not separately evaluate subtypes of leukemia and therefore did not report the subtype-specific risk of leukemia by smoking status. Taken together with our present results, it appears reasonable to say that smoking increases the risk of AML among Asian men. In contrast, associations with ALL and CML among men and women, and with AML in women, were not clear in our study, mainly due to the limited number of events. These should be further assessed in other studies conducted in Asia.

Several mechanisms for the etiologic role of smoking in the development of leukemia have been hypothesized. Various chemicals contained in cigarettes, such as benzene, polonium-210, lead-210, formaldehyde, arsenic, and ammonia, might contribute to direct carcinogenicity.^11^ One of the most promising pathways might be through benzene. Tobacco smoke is known to contain moderately high levels of benzene.^12^ Benzene has been shown to cause chromosomal aberrations, which in turn might be important in the causal pathway of leukemia development.^3^ We were unable to adjust for benzene exposure other than from smoking in this study. However, personal exposure assessment research has indicated that approximately 90% of a smoker's benzene exposure is from smoking.^13^ From this finding, we infer that benzene is an important contributor to smoking-associated leukemia. According to the Surgeon General of the United States, another potential pathway could be through polonium-210 and lead-210 in cigarette smoke.^14^ Ionizing radiation, similar to that emitted by polonium-210 and lead-210 in cigarettes,^15^ has also been implicated in leukemia development.^16^, ^17^, ^18^ Ionizing radiation has the ability to damage DNA, usually by inducing double strand breaks that may cause mutations, deletions, or translocations.^19^ However, Sakoda et al. showed that the annual committed effective dose for Japanese smokers who consumed 20 cigarettes a day corresponded to less than 100 μSv per year,^20^ while epidemiological studies of atomic bomb survivors and other irradiated populations showed an increased cancer risk above 100 mSv.^21^ In contrast, a recent large study of nuclear workers showed that low-dose radiation (mean 1.1 [standard deviation, 2.6] mSv per year) slightly increased leukemia mortality (excess relative risk of leukemia mortality: 2.96 per Gy [90% CI, 1.17–5.21]).^22^ Considering these findings, it is still open to question whether radioactive materials, such as polonium-210 and lead-210, in cigarette smoke could be a true leukemogen. In order to elucidate the biological mechanisms of the leukemogenic effect of smoking, more experimental and epidemiological studies are needed.

The major strengths of our study were its prospective design, high response rate (81%), and negligible proportion of loss to follow-up (0.8%). The collection of information on smoking before cancer diagnosis excluded the exposure recall bias inherent to case–control studies. Several limitations of the study should also be mentioned. First, the statistical power in each category may have been low due to the small number of cases in each category. Overcoming this limitation will require further studies with larger sample sizes. Second, information on smoking was obtained solely on the basis of a single self-report, and no consideration was given to any subsequent change in smoking habit. Therefore, some of the results in this study might not be statistically significant because misclassification of the self-reported smoking habit would probably be nondifferential and may underestimate the true relative risk. Third, lack of information on occupational exposure to high doses of benzene, ionizing radiation, or other carcinogens is another limitation of this study. Finally, we cannot completely rule out the effects of residual confounding by unmeasured variables. Even with adjustment of age at baseline, the Cox model with follow-up length as a time variable for long-term follow-up data could yield a biased estimate.^23^ In addition, the estimates in this study changed when we further adjusted for BMI and occupation. Similarly, some residual confounding by other lifestyle and unknown risk factors might remain.

In conclusion, our results suggest that cigarette smoking increases the risk of AML in Japanese men. In contrast, associations between smoking and AML among women and CML and ALL among men and women were not clear. Similar evaluation in different Asian populations is warranted. Any effort against smoking is important in the prevention of AML, as it is in the prevention of other smoking-associated cancers.

## Conflicts of interest

Manami Inoue is the beneficiary of a financial contribution from the AXA Research fund as chair holder of the AXA Department of Health and Human Security, Graduate School of Medicine, The University of Tokyo. The AXA Research Fund had no role in the design of the study, data collection, analysis, interpretation or manuscript drafting, or in the decision to submit the manuscript for publication.

## Appendix

Members of the Japan Public Health Center-based Prospective Study (JPHC Study, principal investigator: S. Tsugane) Group are: S. Tsugane, N. Sawada, M. Iwasaki, S. Sasazuki, T. Yamaji, T. Shimazu and T. Hanaoka, National Cancer Center, Tokyo; J. Ogata, S. Baba, T. Mannami, A. Okayama, and Y. Kokubo, National Cerebral and Cardiovascular Center, Osaka; K. Miyakawa, F. Saito, A. Koizumi, Y. Sano, I. Hashimoto, T. Ikuta, Y. Tanaba, H. Sato, Y. Roppongi, T. Takashima and H. Suzuki, Iwate Prefectural Ninohe Public Health Center, Iwate; Y. Miyajima, N. Suzuki, S. Nagasawa, Y. Furusugi, N. Nagai, Y. Ito, S. Komatsu and T. Minamizono, Akita Prefectural Yokote Public Health Center, Akita; H. Sanada, Y. Hatayama, F. Kobayashi, H. Uchino, Y. Shirai, T. Kondo, R. Sasaki, Y. Watanabe, Y. Miyagawa, Y. Kobayashi, M. Machida, K. Kobayashi and M. Tsukada, Nagano Prefectural Saku Public Health Center, Nagano; Y. Kishimoto, E. Takara, T. Fukuyama, M. Kinjo, M. Irei, and H. Sakiyama, Okinawa Prefectural Chubu Public Health Center, Okinawa; K. Imoto, H. Yazawa, T. Seo, A. Seiko, F. Ito, F. Shoji and R. Saito, Katsushika Public Health Center, Tokyo; A. Murata, K. Minato, K. Motegi, T. Fujieda and S. Yamato, Ibaraki Prefectural Mito Public Health Center, Ibaraki; K. Matsui, T. Abe, M. Katagiri, M. Suzuki, and K. Matsui, Niigata Prefectural Kashiwazaki and Nagaoka Public Health Center, Niigata; M. Doi, A. Terao, Y. Ishikawa, and T. Tagami, Kochi Prefectural Chuo-higashi Public Health Center, Kochi; H. Sueta, H. Doi, M. Urata, N. Okamoto, F. Ide, H. Goto and R Fujita, Nagasaki Prefectural Kamigoto Public Health Center, Nagasaki; H. Sakiyama, N. Onga, H. Takaesu, M. Uehara, T. Nakasone and M. Yamakawa, Okinawa Prefectural Miyako Public Health Center, Okinawa; F. Horii, I. Asano, H. Yamaguchi, K. Aoki, S. Maruyama, M. Ichii, and M. Takano, Osaka Prefectural Suita Public Health Center, Osaka; Y. Tsubono, Tohoku University, Miyagi; K. Suzuki, Research Institute for Brain and Blood Vessels Akita, Akita; Y. Honda, K. Yamagishi, S. Sakurai and N. Tsuchiya, University of Tsukuba, Ibaraki; M. Kabuto, National Institute for Environmental Studies, Ibaraki; M. Yamaguchi, Y. Matsumura, S. Sasaki, and S. Watanabe, National Institute of Health and Nutrition, Tokyo; M. Akabane, Tokyo University of Agriculture, Tokyo; T. Kadowaki and M. Inoue, The University of Tokyo, Tokyo; M. Noda and T. Mizoue, National Center for Global Health and Medicine, Tokyo; Y. Kawaguchi, Tokyo Medical and Dental University, Tokyo; Y. Takashima and Y. Yoshida, Kyorin University, Tokyo; K. Nakamura and R. Takachi, Niigata University, Niigata; J. Ishihara, Sagami Women's University, Kanagawa; S. Matsushima and S. Natsukawa, Saku General Hospital, Nagano; H. Shimizu, Sakihae Institute, Gifu; H. Sugimura, Hamamatsu University School of Medicine, Shizuoka; S. Tominaga, Aichi Cancer Center, Aichi; N. Hamajima, Nagoya University, Aichi; H. Iso and T. Sobue, Osaka University, Osaka; M. Iida, W. Ajiki, and A. Ioka, Osaka Medical Center for Cancer and Cardiovascular Disease, Osaka; S. Sato, Chiba Prefectural Institute of Public Health, Chiba; E. Maruyama, Kobe University, Hyogo; M. Konishi, K. Okada, and I. Saito, Ehime University, Ehime; N. Yasuda, Kochi University, Kochi; S. Kono, Kyushu University, Fukuoka; S. Akiba, Kagoshima University, Kagoshima; T. Isobe, Keio University, Tokyo; Y. Sato, Tokyo Gakugei University, Tokyo.
